# Influence of daily beer or ethanol consumption on physical fitness in response to a high-intensity interval training program. The BEER-HIIT study

**DOI:** 10.1186/s12970-020-00356-7

**Published:** 2020-05-27

**Authors:** Cristina Molina-Hidalgo, Alejandro De-la-O, Manuel Dote-Montero, Francisco J. Amaro-Gahete, Manuel J. Castillo

**Affiliations:** EFFECTS 262 Department of Physiology, Faculty of Medicine, University of Granada, 18016 Granada, Spain

**Keywords:** Exercise, Alcohol, Fitness, Strength, VO_2_max

## Abstract

**Background:**

High-intensity interval training (HIIT) is an effective approach to improve physical fitness, but consuming beer, which is a regular practice in many physically active individuals, may interfere with these effects. The purposes of this study were to investigate the effects of a 10-week (2 days/week) HIIT program on cardiorespiratory fitness, muscle strength and power parameters, and also to assess the possible influence on them of a moderate consumption of beer (at least from Monday to Friday) or its alcohol equivalent.

*Methods:* Young (24 ± 6 years old) healthy adults (*n* = 73, 35 females) were allocated to five groups. Four groups participated in the HIIT intervention program while the fifth group was a control Non-Training group (*n* = 15). Participants in the training groups chose whether they preferred receiving alcohol or alcohol-free beverages. Those choosing alcohol were randomized to either beer or ethanol intake: (i) T-Beer group (alcohol beer, 5.4%; *n* = 13) or (ii) T-Ethanol (sparkling water with vodka, 5.4%; *n* = 14). Those choosing alcohol-free intake were randomized to (iii) T-Water group (sparkling water, 0.0%; *n* = 16), or (iv) T-0.0Beer group (alcohol-free beer, 0.0%; *n* = 15). Men ingested 330 ml of the beverage at lunch and 330 ml at dinner; women ingested 330 ml at dinner. Before and after the intervention, maximal oxygen uptake in absolute and relative terms (VO_2_max.), maximal heart rate, total test duration, hand grip strength and four types of vertical jumps were measured.

**Results:**

HIIT induced significant improvements in absolute and relative values of VO_2_max, and total test duration (all *p* < 0.05) in all the training groups; also, clinical improvements were found in hand grip strength. These positive effects were not influenced by the regular intake of beer or alcohol. No changes in the vertical jumps occurred in any of the groups.

**Conclusions:**

A moderate beer or alcohol intake does not mitigate the positive effect of a 10-week HIIT on physical fitness in young healthy adults.

**Trial registration:**

ClinicalTrials.gov ID: NCT03660579. Registered 20 September 2018. Retrospectively registered.

## Background

Physical fitness is the ability to do a physical activity and/or physical exercise using most of the body structures [1, 2]. Physical fitness integrates several components [1], such as cardiorespiratory fitness and muscular strength, which are widely recognized as powerful markers of sport performance, health-related outcomes and relevant determinants of current and future health status as well as important predictors of all-cause mortality [1, 3, 4]. Emerging evidence suggests that high-intensity interval training (HIIT), which involves repeated bouts (< 45 s) of intense exercise (85–95% maximal heart rate) interspersed with periods of rest or lower-intensity active recovery, is an effective strategy to get important improvements in cardiorespiratory fitness [5–9] and muscular strength [5–10]. Furthermore, HIIT overcomes one of the most common personal barriers to physical exercise training as it is the lack of time, since HIIT is a less time-consuming training methodology which allows to maximize the potential benefits induced by exercise [11, 12].

After training, optimal resting and adequate nutrition are crucial to allow not only the recovery of energy reserves, but also the removal, regeneration, repair and protection of damaged or worn structures [13, 14]. This brings the functional structures to a state of adaptation with supercompensation, such as an increased skeletal muscle oxidative capacity, increased resting glycogen content, a reduced rate of glycogen utilization and lactate production, or increased capacity for whole-body and skeletal muscle lipid oxidation, among others. These adaptations improve exercise performance, promote health as well as well-being [15–18] and represent the physiological basis of the multiple and positive benefits of moderate-to-vigorous exercise training [7].

One common post-exercise nutritional practice is the consumption of beer, which is one of the most consumed beverages in the world [19, 20] and is frequently consumed by adults after exercise in Western countries [21]. Actually, consumption of beer and, more rarely, other alcohol-containing beverages is often encouraged as a social aspect of sport activities, particularly in the case of recreational contexts, or to facilitate team bonding as part of a post-match celebration and relaxation [22, 23]. Beer content is mainly water (90%) but also contains a certain amount of alcohol (range approximately from 3.5 to 7%) [24]. There is a concern about the effects that beer can have on hydration, recovery, and exercise performance due to the effects of alcohol [25]. These negative effects can be associated with a reduced production of testosterone and its subsequent effects on protein synthesis and muscular adaptation/regeneration, and with an increase in the diuretic response of the body due to the inhibitory effect of ethanol on vasopressin release [26–28]. Even though the damages associated with a high intake of alcohol have been widely studied and are beyond doubt [28], the effects of moderate doses remain under debate [24, 29]. Some authors conclude that alcohol consumed after an exercise session could interfere with some aspects of the performance, such as strength recovery [28]. However, a low dose of alcohol seems not to affect strength recovery [30], neither power recovery in men [31].

The differences in exercise modalities, alcohol doses, types of beverages and participant characteristics, including their habitual alcohol intake, may explain the contradictory and inconclusive results found among studies [28]. However, there are no studies that investigate the influence of a moderate consumption of beer or alcohol on the physical fitness response to a highly demanding training program in conditions reflecting the real-life situation of healthy adults. Thus, the purpose of this study was to evaluate the effects of a HIIT program on physical fitness parameters in healthy young adults, and to analyze the possible influence of daily but moderate beer consumption or its alcohol equivalent on it. The primary hypothesis was that HIIT would improve cardiorespiratory fitness, muscular strength and power outcomes, but the intake of beer, even in moderate amounts, may mitigate these positive effects due to its alcohol content.

## Methods

### Study design and participants

Following recruitment via social networks, local media, and posters, a total of 83 healthy adults (35 females) from Granada (Spain) were assessed for eligibility. This study is a registered controlled trial (ClinicalTrials.gov ID: NCT03660579) and follows the latest revision of the Helsinki Declaration. The Ethics Committee on Human Research of the University of Granada (321/CEIH/2017) approved the study design, the study protocols, and the informed consent procedure. Prior to the enrolment, all potential individuals completed a medical examination to identify any pathological condition, provided a written informed consent, and were fully informed about the study objectives, design, inclusion criteria, assessments to be undertaken, exercise program intervention, and types of beverages to be ingested. The inclusion criteria were as follows: (i) having a body mass index (BMI) from 18.5 to 30 kg/m^2^, (ii) not being engaged in a concurrent or previous structured training program or a weight-loss program, (iii) having a stable body mass during the last 5 months (body mass changes < 3 kg), (iv) being free of disease, (v) not being pregnant or lactating; (vi) not taking any medication for chronic diseases, and (vii) not suffering pain, recent injuries, or other problems preventing strenuous physical activity. All participants were informed about the physical activity recommendations of the World Health Organization during the intervention program, as well as the benefits of practicing physical activity [32], and they were instructed to maintain their usual physical activity levels and not to engage in other additional structured exercise outside of the intervention program.

**Fig. 1:**
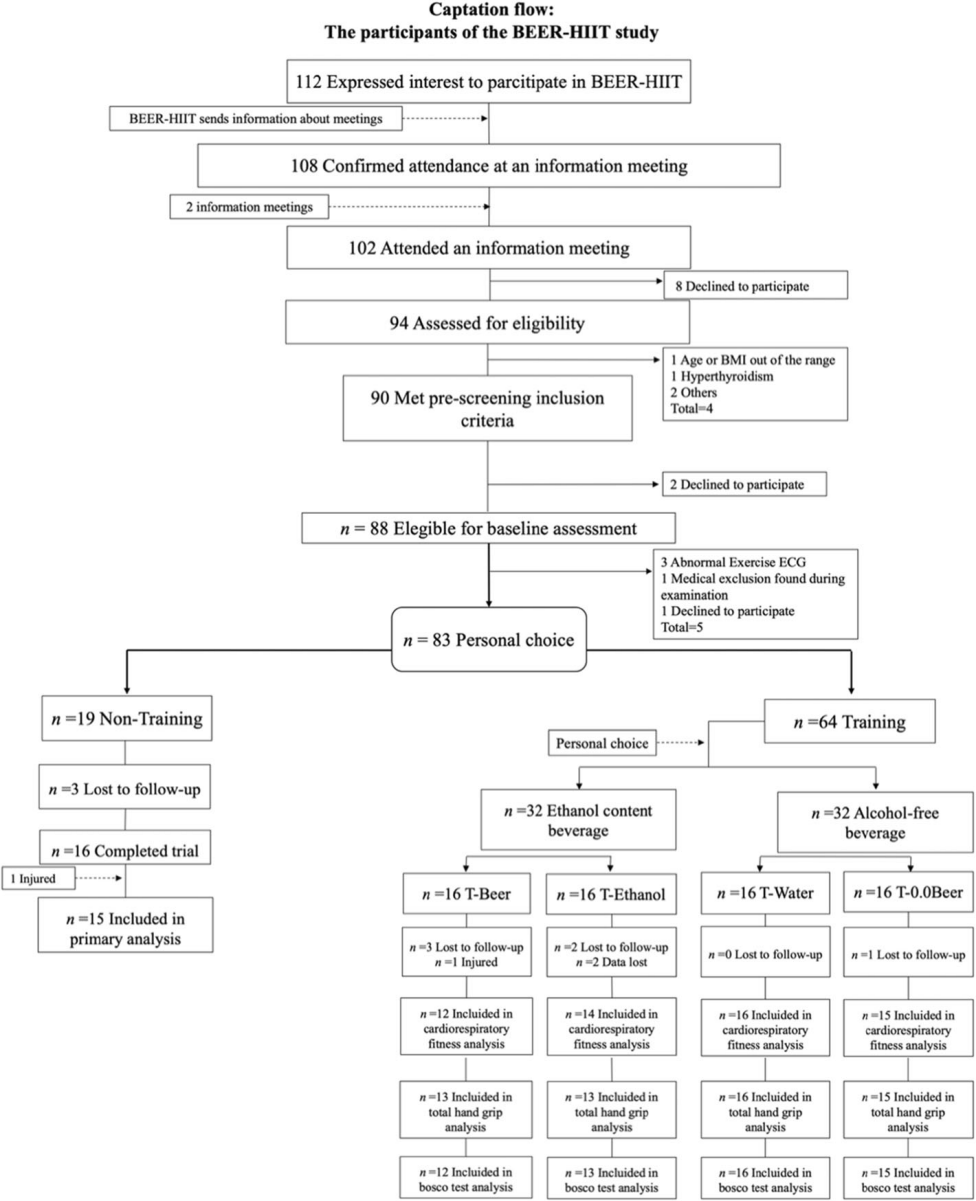
Flow-chart diagram

As shown in Fig. 1, 94 individuals attended an information meeting and were assessed for eligibility. A total of 83 individuals met the inclusion criteria and, after the baseline measurements, the participants were allocated to a training (T) or a Non-Training group based on personal preferences. Those allocated to the T group then chose whether they preferred to be included in a group ingesting an ethanol-containing beverage (5.4% alcohol content) or an alcohol-free beverage from Monday to Friday. Participants choosing ethanol were randomly allocated to either a group consuming beer (T-Beer) or to a group consuming sparkling water with added vodka ethanol (T-Ethanol). Those choosing non-alcoholic beverages were randomly allocated to either an alcohol-free beer group (T-0.0Beer) or to a sparkling-water group (T-Water). Each group was composed of 8 men and 8 women. This type of non-random (based on individual preference) and random allocation of the participants was conducted following ethical considerations and advice suggested by the ethical committee (321-CEIH-2017), since drinking alcohol, or participating in a highly demanding training program should be a personal choice. The assessment staff was blinded to the participants’ randomization assignment.

The participants were asked to report their usual frequency of alcohol intake in seven possible response categories using the Beverage Intake Questionnaire (BEVQ [26]) before and after the intervention. This questionnaire was developed to estimate mean daily intake of water, sugar-sweetened beverages, and alcohol beverages. To score the BEVQ, frequency (“How often”) is converted to the unit of times per day, and then multiplied by the amount consumed (“How much each time”) to provide average daily and weekly intake beverage consumption. Total alcohol consumption was quantified through the sum of beverage categories containing alcohol (i.e. beer, wine, spirits, and cocktails). In addition, the physical activity levels were registered before and after the intervention program by self-report. A total of 10 participants dropped out between the allocation and the follow-up due to (i) lack of adherence to the training program (*n* = 5), (ii) physical reasons (*n* = 3), or (iii) other reasons (*n* = 2).

### Training protocol

A detailed description of each exercise of the training program can be found elsewhere and the proposed exercises are based on our previous experience [33, 34]. Attending at least 80% of sessions was required to be included in the final analysis. All training sessions were supervised by qualified and certified personal trainers and performed in groups of ~ 8 participants. An intensity gradual progression was also scheduled in order to ensure a good adherence of the intervention group.

The HIIT intervention groups completed a total of 2 sessions per week in the late afternoon or early evening from Monday to Friday for 10 weeks with at least 48 h of recovery between each session. A gradual progression was proposed to avoid injuries and drop outs. Training sessions started with a volume of 40 min per week and an intensity of 8–9 RPE following previous studies [33, 35, 36]. Subsequent increments of both volume and intensity were established in Phase I (50 min per week and 10 RPE) and in Phase II (65 min per week and 10 RPE).

The HIIT intervention was divided into two different phases, starting with a familiarization phase to learn the main movement patterns. In all cases, the intensity of the training was > 8 Rating of Perceived Exertion (0–10 RPE scale) [37], which has a positive linear relationship with heart rate and VO_2_max (maximal oxygen uptake) [38, 39]. There was a passive rest between exercises and an active rest between sets (an intensity of 6 RPE, which corresponds with 60% VO_2_max [38, 40]), following the periodization described in previous studies [41]. Eight self-loading exercises were performed in a circuit form twice per set (i.e., frontal plank, high knees up, TRX horizontal row, squat, deadlift, side plank, push up, and burpees) with a passive rest between exercises and an active rest between sets (6 RPE intensity).

A dynamic standardized warm up and an active global-stretching cooling-down protocols were completed at the beginning and at the end of each training session, respectively, in all intervention groups. An extra effort was made to elicit better adherence to the training protocol. For instance, the sessions were rescheduled when a participant was unable to attend due to work, personal issues, or illness. The participants were constantly encouraged throughout each training session and were instructed to reach the specific target intensity.

### Beverage intake protocol

The volumes of ingested fluid were 660 ml for men and 330 ml for women from Monday to Friday during the 10-week intervention. Men ingested 330 ml at lunch and 330 ml at dinner, and women ingested 330 ml at dinner: (i) the T-Beer group ingested regular Lager Beer (5.4% alcohol-Alhambra Especial®, Granada, Spain); (ii) the T-0.0Beer group ingested alcohol-free beer (0.0% alcohol-Cruzcampo®, Sevilla, Spain); (iii) the T-Water group ingested sparkling water (Eliqua 2®, Font Salem, Spain); (iv) the T-Ethanol group ingested sparkling water with exactly the equivalent amount of distilled alcohol added. The distilled alcoholic beverage used in our study was branded vodka because of the purity of its composition (37.5% ethanol and 62.5% water). The amount of alcohol selected to ingest was based on scientific evidence, which defines a moderate amount as two or three drinks/day or 24–36 g of ethanol/day for men and one to two drinks/day or 12–24 g of ethanol/day for women [28, 36]. The only alcohol consumption allowed from Monday to Friday was that provided by the investigators. During the weekend, those participants included in alcohol groups were requested to drink no more than a moderate amount of alcohol (i.e., 660 ml/day for men and 330 ml/day for women). All the beverages were coded and provided by a staff member of our research laboratory at the beginning of each week to maintain blinded the group assignment of the participants to the investigators who did the evaluations. Additionally, the alcohol intakes were registered before and after the intervention.

### Physical fitness assessment

#### Cardiorespiratory fitness

A maximum treadmill (H/P/Cosmos Pulsar treadmill, H/P/ Cosmos Sport & Medical GMBH, Germany) exercise test applying a previously validated protocol [33, 35, 42] (i.e., the modified Balke protocol [43]) was used to determine VO_2_max. We conducted a warm up (walking at 3.5 km/h for 1 min and at 4 km/h for 2 min) followed by an incremental protocol which maintained a speed of 5.3 km/h at 0% grade for 1 min. Participants walked on a treadmill at a constant speed (5.3 km/h) with increases of 1% every minute until volitional exhaustion. We measured O_2_ uptake and CO_2_ production with an indirect calorimeter using an oronasal mask (model 7400, Hans Rudolph Inc., Kansas City, MO, United States) equipped with a prevent TM metabolic flow sensor (Medgraphics Corp., MN, United States). We calibrated the gas analyzer using two standard gas concentrations and we performed a flow calibration with a 3-L calibration syringe before the test every day. The Breeze Suite software (version 8.1.0.54 SP7, MGC Diagnostic®) was used to assess averages for oxygen uptake (VO_2_) and respiratory exchange ratio (the ratio between carbon dioxide and oxygen percentage) every minute. In addition, heart rate was continuously measured throughout the exercise test (Polar RS800, Kempele, Finland), and ratings of perceived exertion (RPE) were assessed at each stage and at exhaustion (during the last 15 s) using the 6–20 Borg scale [44]. A familiarization process with the RPE scale was conducted before the exercise test. During each trial, the participants were strongly encouraged to make their utmost effort. The criteria for achieving VO_2_max were: (i) a respiratory exchange ratio ≥ 1.1, a plateau in VO_2_ /change of < 100 ml/min in the last three consecutive 10 s stage), (ii) a heart rate within 10 beats/min of the aged predicted maximal heart rate (168–0.7*age) [45, 46], and (iii) if these criteria were not met, the peak oxygen uptake value during the exercise test was considered [46].

The participants were asked to refrain from stimulant substances and from ingesting food 24 h and 2 h before the exercise test, respectively, and not to perform any physical activity of moderate (previous 24 h) and/or vigorous intensity (previous 48 h).

#### Muscular strength

Hand grip strength (kg) was assessed with a digital hand dynamometer (T.K.K. 5401 Grip-D; Takey, Tokyo, Japan). This measurement has been suggested as a valid test to predict muscular strength and endurance with a simpler equipment and minimizing efforts from subjects. Also, it has been associated with whole body and upper body strength [47], although greater benefits for strength improvements would have been obtained by using a battery of strength tests such as those used in the weight-bearing exercises practiced during the training sessions. Participants maintained a standard bipedal position during the entire test with the arm in complete extension preventing the dynamometer from coming in contact with any part of the body except the hand being measured. Two attempts were made for each hand, with a 1-min rest between each trial. We instructed the participants to continuously squeeze for 2–3 s and they were asked to exert their maximal force in every attempt. Following previous studies, we fixed the grip spam of the dynamometer at 5.5 cm for men and a validated equation was used for women [48]. We considered total hand grip strength as the sum of the best attempt for both the left and right hand.

#### Muscular power

The anaerobic power of lower limbs was evaluated through the “Ergo Jump Bosco System®” (Globus, Treviso, Italy). The participants performed two trials for each jumping exercise and the best score was recorded [49]. Four types of jumps were performed in the following order: squat jump, SJ; countermovement jump without arm-swing, CMJ; with arm-swing, Abalakov jump, ABKJ; and drop jump, DJ. The participants received specific feedback from the investigators indicating leg, arm and trunk position during the test. The participants performed some practice trials before the assessment [50]. The results from the above-mentioned tests allowed the calculation of relevant muscle-strength-related indexes: elasticity index (elastic energy = ({CMJ-SJ}/CMJ)× 100), upper limbs coordination index ((ABKJ-CMJ)/ABKJ)× 100), and percentage of fast-twitch fibers [51].

### Statistical analysis

Sample size calculations were based on a minimum predicted change of 15% in VO_2_max, hand grip strength and SJ jump (with a 15% of estimated standard deviation) between the intervention groups and the control Non-Training group. Considering the results of a pilot study, 13 participants per group were necessary to provide a statistical power of 85% (type I error = 0.05) [52]. However, we recruited a minimum of 16 participants per group (a total of 80) to accept a maximum loss of 20% at the follow up [53].

Data were checked for normality using a Shapiro–Wilk test and a visual inspection of Q-Q plots. We conducted repeated measure analyses of variance to study changes in cardiorespiratory fitness and muscular strength parameters (i.e. VO_2_max. in absolute and relative terms, maximal heart rate, total test duration, total hand grip, SJ, CMJ, ABKJ and DJ) across time, between groups, and their interaction (time × group). We applied the *Student’s t-test* to paired values to determine intragroup differences in cardiorespiratory fitness and muscular strength parameters before and after the 10-week intervention.

An analysis of covariance (ANCOVA) was conducted to determine the effect of the groups (fixed factor) on cardiorespiratory fitness and muscular strength outcomes, i.e., post-VO_2_max. minus pre- VO_2_max. (dependent variable), adjusting for the baseline values (model 1). We conducted the same analysis for changes in VO_2_max. in relative terms, maximal heart rate, total test duration, total hand grip, SJ, CMJ, ABKJ and DJ. Bonferroni post hoc tests with adjustment for multiple comparisons. Additional models were conducted controlling for baseline values and sex (model 2) and for baseline values and age (model 3) (see Additional file 1).

The level of statistical significance was defined at *p* < 0.05. The statistical analyses were conducted in Statistical Package for Social Science (SPSS, V. 25.0, IBM SPSS Statistics, IBM Corporation), and the graphical plots were conducted in GraphPad Prism 5 (GraphPad Software, San Diego, CA, USA).

## Results

A total of 73 participants (35 women) were included in the analyses after a loss to follow up of 12% (see Fig. 1).

BMI, body mass index; ECG, electrocardiogram; T-Beer, the group that performed HIIT and consumed alcohol beer; T-0.0Beer, the group that performed HIIT and consumed non-alcoholic beer; T-Water, the group that performed HIIT and consumed sparkling water; T-Ethanol, the group that performed HIIT and consumed sparkling water with alcohol added.

The characteristics of the study population by sex are shown in Table 1. No differences were observed in the baseline values, neither in the training background existed between groups. The reported intakes of alcohol in the different groups were also similar (*p* = 0.144; See Table 1). The distribution of the number of men and women was nearly equal in each group.

**Table 1:** Descriptive parameters before the intervention program

	Non-Training (***n*** = 15)		T-Beer (***n*** = 13)		T-0.0Beer (***n*** = 15)		T-Water (***n*** = 16)		T-Ethanol (***n*** = 14)	
	Men (***n*** = 8)	Women (***n*** = 7)	Men (***n*** = 6)	Women (***n*** = 7)	Men (***n*** = 8)	Women (***n*** = 7)	Men (***n*** = 9)	Women (***n*** = 7)	Men (***n*** = 7)	Women (***n*** = 7)
Age (years)	19.9 ± 2.3	20.1 ± 2.9	25.2 ± 5.5	23.9 ± 8.1	25.4 ± 8.1	23.4 ± 4.0	26.9 ± 7.6	20.9 ± 2.7	26.9 ± 7.0	25.3 ± 6.8
Body Mass (kg)	72.0 ± 9.8	57.3 ± 4.5	80.3 ± 16.3	61.3 ± 8.5	82.3 ± 15.3	59.2 ± 7.4	73.6 ± 6.3	64.5 ± 12.3	77.9 ± 13.3	58.8 ± 7.8
Body Mass Index (kg/m^2^)	22.7 ± 2.2	21.4 ± 1.2	26.4 ± 4.4	22.0 ± 3.2	26.8 ± 3.4	22.9 ± 2.9	25.6 ± 2.9	23.8 ± 3.9	26.2 ± 3.9	22.2 ± 3.5
Weekly Alcohol Intake (liters)	0.9 ± 0.9	1.1 ± 0.6	0.2 ± 0.5	0.9 ± 0.9	1.0 ± 0.9	2.0 ± 1.4	1.0 ± 1.1	0.9 ± 1.1	1.1 ± 1.2	0.5 ± 0.6
*Cardiorespiratory fitness*	**Men (*n*** **= 7)**	**Women (*n*** **= 7)**	**Men (*n*** **= 6)**	**Women (*n*** **= 6)**	**Men (*n*** **= 8)**	**Women (*n*** **= 7)**	**Men (*n*** **= 9)**	**Women (*n*** **= 7)**	**Men (*n*** **= 7)**	**Women (*n*** **= 7)**
VO_2_ max. (ml/min)	3091 ± 515	2144 ± 297	3230 ± 419	2232 ± 382	3154 ± 587	2219 ± 317	2699 ± 296	2168 ± 83	2971 ± 516	2137 ± 299
VO_2_ max. (ml/kg/min)	44 ± 10	37 ± 6	41 ± 8	35 ± 4	38 ± 5	37 ± 4	37 ± 6	35 ± 5	38 ± 4	36 ± 3
Maximal heart rate (b/min)	188 ± 13	190 ± 14	188 ± 18	188 ± 10	185 ± 7	189 ± 12	177 ± 13	184 ± 12	181 ± 14	187 ± 9
Total test duration (s)	1207 ± 321	1041 ± 175	1231 ± 295	1035 ± 108	1074 ± 136	1025 ± 150	1086 ± 212	966 ± 151	1129 ± 224	1006 ± 145
*Muscular Strength*	**Men (*n*** **= 8)**	**Women (*n*** **= 7)**	**Men (*n*** **= 6)**	**Women (*n*** **= 7)**	**Men (*n*** **= 8)**	**Women (*n*** **= 7)**	**Men (*n*** **= 9)**	**Women (*n*** **= 7)**	**Men (*n*** **= 6)**	**Women (*n*** **= 7)**
Total hand grip (kg)	73.5 ± 12.5	59.9 ± 6.1	82.2 ± 13.7	57.6 ± 6.4	78.2 ± 19.0	50.5 ± 6.3	75.8 ± 21.1	51.1 ± 8.4	85.1 ± 20.7	56.7 ± 6.4
*Muscular Power*	**Men (*n*** **= 7)**	**Women (*n*** **= 7)**	**Men (*n*** **= 6)**	**Women (*n*** **= 6)**	**Men (*n*** **= 8)**	**Women (*n*** **= 7)**	**Men (*n*** **= 9)**	**Women (*n*** **= 7)**	**Men (*n*** **= 6)**	**Women (*n*** **= 7)**
Squat Jump (cm)	29.8 ± 6.3	21.4 ± 5.3	29.5 ± 3.5	20.9 ± 6.9	26.9 ± 10.5	19.6 ± 2.8	26.7 ± 7.0	19.9 ± 6.5	27.5 ± 7.7	22.9 ± 5.2
Counter-movement Jump (cm)	32.0 ± 8.1	21.8 ± 4.4	31.1 ± 1.9	22.1 ± 6.2	24.3 ± 4.4	18.5 ± 1.4	28.4 ± 8.9	20.5 ± 7.7	30.0 ± 7.5	20.0 ± 2.0
Abalakov Jump (cm)	35.4 ± 7.6	24.2 ± 5.2	36.9 ± 4.3	24.1 ± 6.1	31.6 ± 10.1	22.4 ± 4.2	34.1 ± 9.7	23.9 ± 7.4	35.8 ± 10.5	25.8 ± 3.5
Drop Jump (cm)	36.3 ± 8.1	23.0 ± 5.1	35.2 ± 3.1	25.4 ± 5.8	28.7 ± 4.8	21.1 ± 2.7	33.4 ± 9.0	22.9 ± 7.8	37.0 ± 14.9	26.3 ± 5.6

Data are shown as mean ± standard deviation. T-Beer, the group that performed HIIT and consumed alcohol beer; T-0.0Beer, the group that performed HIIT and consumed non-alcoholic beer; T-Water, the group that performed HIIT and consumed sparkling water; T-Ethanol, the group that performed HIIT and consumed sparkling water with alcohol added.

**Fig. 2:**
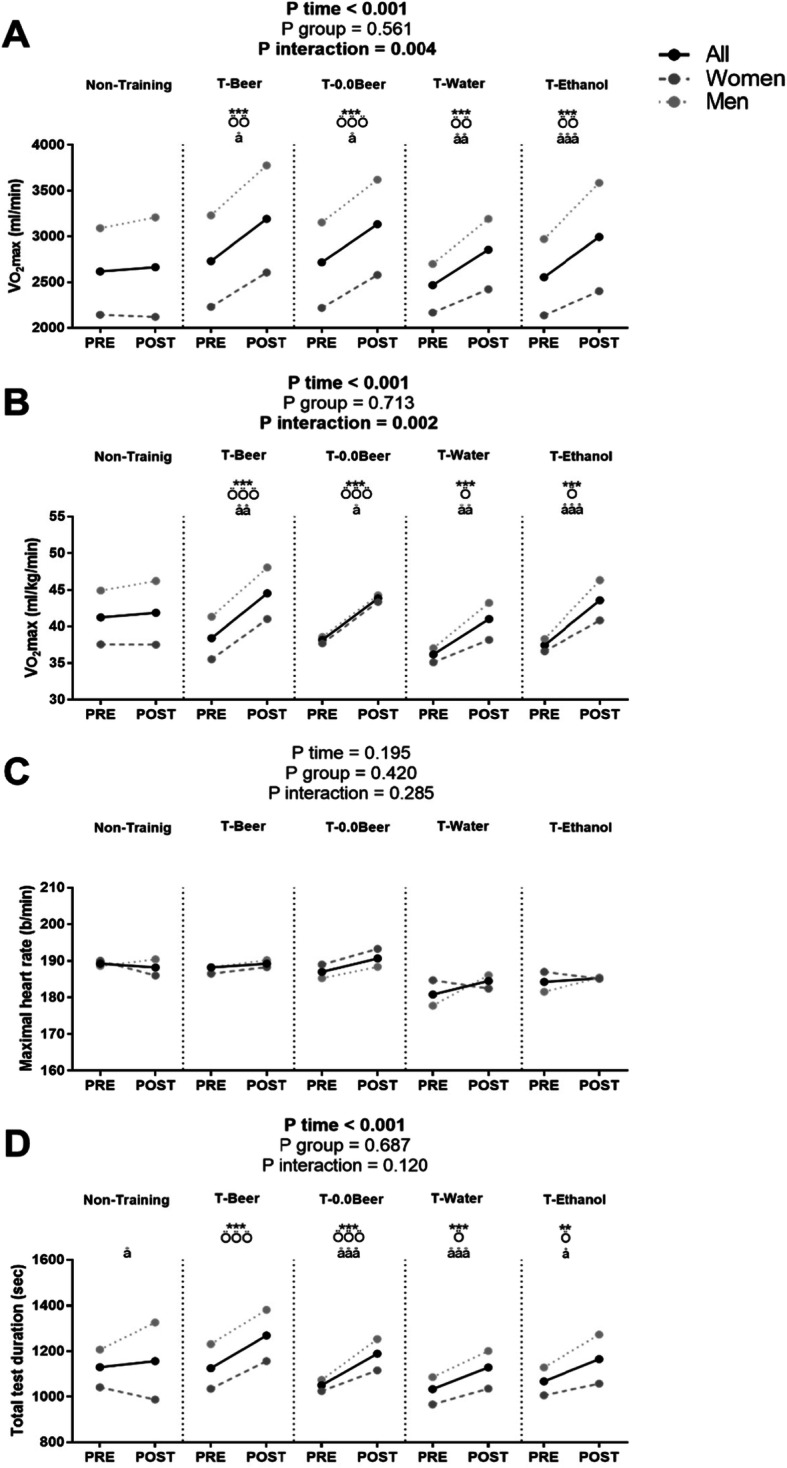
Changes in maximum oxygen uptake (VO_2_max.) in absolute (**a**) and relative terms (**b**), maximal heart rate (**c**), and total test duration (**d**) before and after the intervention study. *P*-value [time, group, and interaction (time x group)] of repeated measure analysis of variance. ** *p* < 0.01, *** *p* < 0.001 for total sample, ö *p* < 0.05, öö *p* < 0.01, ööö, *p* < 0.001 for women, å *p* < 0.05, åå *p* < 0.01, ååå *p* < 0.001 for men, obtained by Student’s paired t-test. Data are shown as means. Abbreviations: T-Beer, group that performed HIIT and consumed alcohol beer; T-0.0Beer, the group that performed HIIT and consumed non-alcoholic beer; T-Water, the group that performed HIIT and consumed sparkling water; T-Ethanol, the group that performed HIIT and consumed sparkling water with added alcohol

Figure 2 shows cardiorespiratory fitness-related variables before and after the intervention study. A significant time x group interaction was found in VO_2_max. in absolute and relative values (*p* = 0.004 and *p* = 0.002, respectively), whereas no significant time x group interaction was found in maximal heart rate and total test duration (all *p* > 0.05). VO_2_max. in absolute and relative terms increased in all the intervention groups with no influence of the beverage intake (2730 ± 658 vs. 3192 ± 784 ml/min for T-Beer, 2718 ± 670 vs. 3134 ± 757 ml/min for T-0.0Beer, 2466 ± 351 vs. 2855 ± 563 ml/min for T-Water, 2554 ± 593 vs. 2994 ± 709 ml/min for T-Ethanol in absolute VO_2_max.; all *p* < 0.001, Fig. 2a; and 38 ± 7 vs. 44 ± 7 ml/kg/min for T-Beer, 38 ± 4 vs. 43 ± 5 ml/kg/min for T-0.0Beer, 36 ± 5 vs. 41 ± 7 ml/kg/min for T-Water, and 37 ± 4 vs. 43 ± 4 ml/kg/min for T-Ethanol in relative VO_2_max.; all *p* < 0.001, Fig. 2b). Similarly, the total test duration significantly increased in all the intervention groups without influence of the beverage intake (1125 ± 229 vs. 1269 ± 261 s for T-Beer, 1051 ± 140 vs. 1189 ± 155 s for T-0.0Beer, and 1067 ± 192 vs. 1165 ± 211 s for T-Ethanol; all *p* < 0.05, Fig. 2d). No statistical differences were noted in the Non-Training group in any case (all *p* > 0.05).

**Fig. 3:**
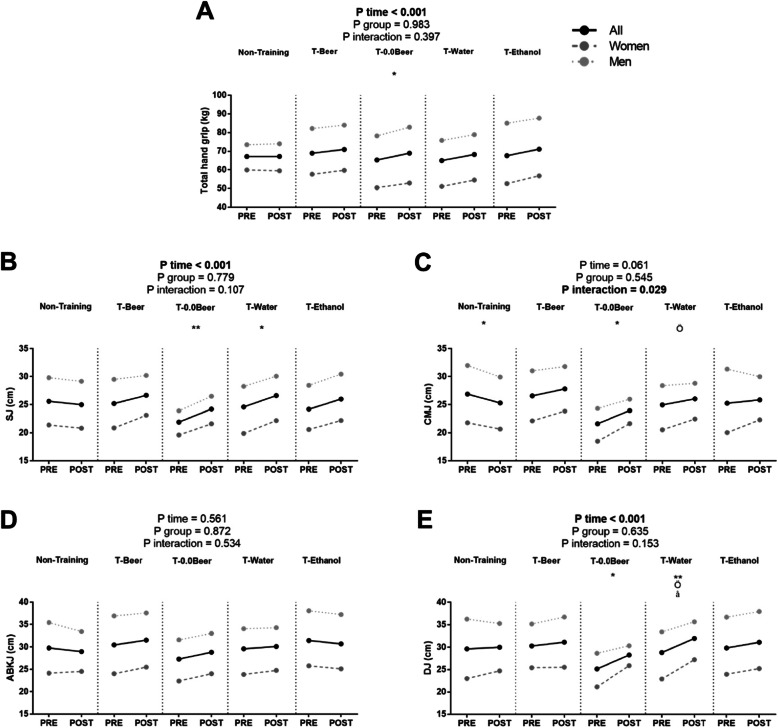
Changes in total hand grip (**a**), squat jump (**b**), counter-movement jump (**c**), Abalakov jump (**d**), and drop jump (**e**) before and after the intervention study. *P*-value [time, group, and interaction (time x group)] of repeated measure analysis of variance. * *p* < 0.05; ** *p* < 0.01 for total sample, ö *p* < 0.05, for women, å *p* < 0.05 for men, obtained by Student’s paired t-test. Data are shown as means. Abbreviations: SJ, squat jump; CMJ, counter-movement jump; ABKJ, Abalakov jump; DJ, drop jump; T-Beer, the group that performed HIIT and consumed alcohol beer; T-0.0Beer, the group that performed HIIT and consumed non-alcoholic beer; T-Water, the group that performed HIIT and consumed sparkling water; T-Ethanol, the group that performed HIIT and consumed sparkling water with alcohol added

Figure 3 shows muscular strength and power-related variables before and after the intervention study. A significance time x group interaction was found in CMJ (*p* = 0.029, Fig. 3), whereas no significance was observed in total hand grip strength, SJ, ABKJ and DJ (all *p* > 0.05, Fig. 3). Total hand grip performance only increased significantly in the T-0.0Beer group (65.3 ± 20.1 vs. 68.9 ± 19.3 kg; *p* = 0.0041, Fig. 3a), whereas no statistically significant improvements were found in the rest of the intervention groups. SJ, CMJ and DJ performance increased in the T-0.0Beer group (21.9 ± 4.7 vs. 24.2 ± 4.4 cm for SJ, 21.6 ± 4.4. vs. 24.0 ± 4.8 cm for CMJ, and 25.2 ± 5.5 vs. 28.3 ± 4.3 cm for DJ; all *p* < 0.05, Fig. 3b, c and d) as well as in the T-Water group (24.6 ± 8.5 vs. 26.6 ± 8.3 cm for SJ, and 28.8 ± 9.8 vs. 32.0 ± 9.3 cm for DJ; all *p* < 0.05, Fig. 3b and e). No statistical differences were noted in the Non-Training group for SJ, ABKJ and DJ (all *p* > 0.05), whereas a significant decrease was observed in CMJ (26.9 ± 8.2 vs. 25.3 ± 7.9 cm; *p* = 0.022, Fig. 3b).

**Fig. 4:**
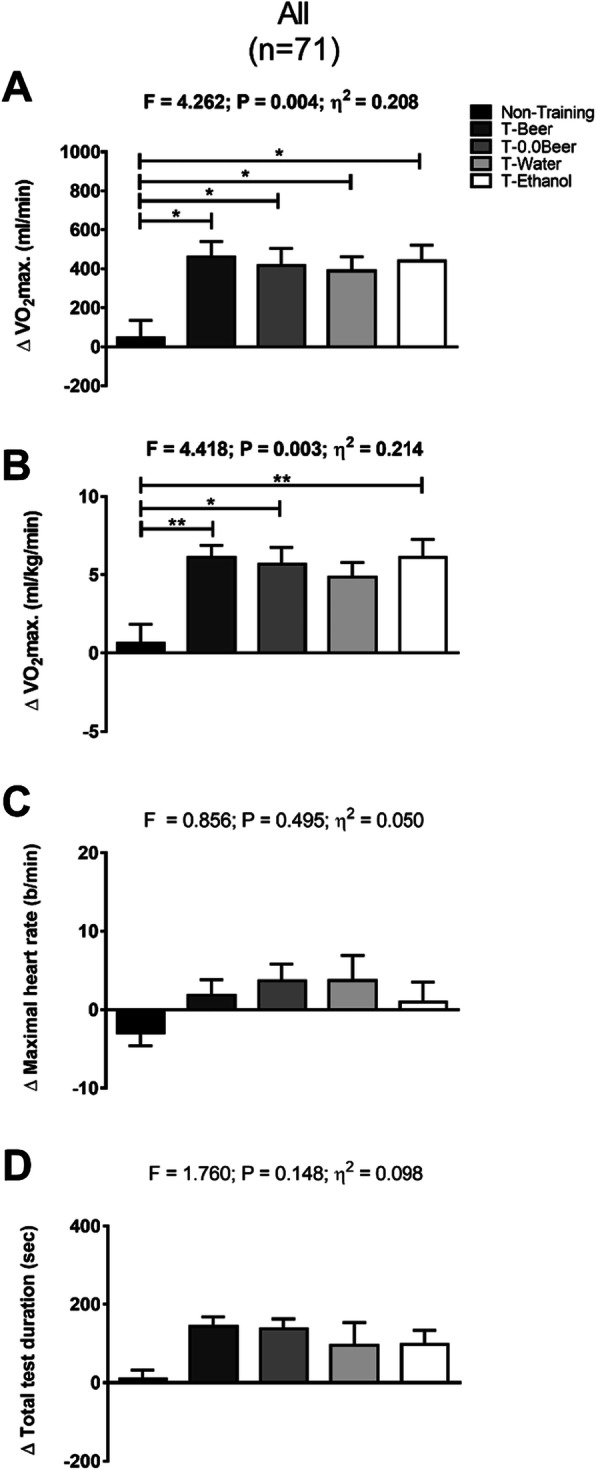
Changes in maximum oxygen uptake (VO2max.) in absolute (**a**) and relative terms (**b**), maximal heart rate (**c**), and total test duration (**d**), after the intervention study between the five groups. Significant differences between groups applying an analysis of covariance adjusting for baseline values with post hoc Bonferroni-corrected t-test are indicated as: * *p* < 0.05 ** *p* < 0.01, *** *p* < 0.001. Data are shown as means ± standard error of the mean. Abbreviations: ɳ2, partial eta squared; T-Beer, the group that performed HIIT and consumed alcohol beer; T-0.0Beer, the group that performed HIIT and consumed non-alcoholic beer; T-Water, the group that performed HIIT and consumed sparkling water; T-Ethanol, the group that performed HIIT and consumed sparkling water with alcohol added

Figure 4 shows changes in the cardiorespiratory fitness-related variables after the intervention study between the five groups (total, men, and women). The T-Beer, the T-0.0Beer, the T-Water, and the T-Ethanol intervention groups similarly increased VO_2_max. in absolute and relative terms compared with the Non-Training group (all *p* < 0.05), with no significant differences between them (all *p* > 0.05). The results persisted in the T-Beer and the T-0.0Beer women groups after analyzing data divided by sex (see Additional file 1B and 1D). All intervention groups showed similar increases in the total test duration compared with the Non-Training group, with statistical differences between the T-Beer women group vs. the Non-Training women group (see Additional file 1H). The results persisted when the analyses were additionally adjusted for sex and age (see Additional file 3).

**Fig. 5:**
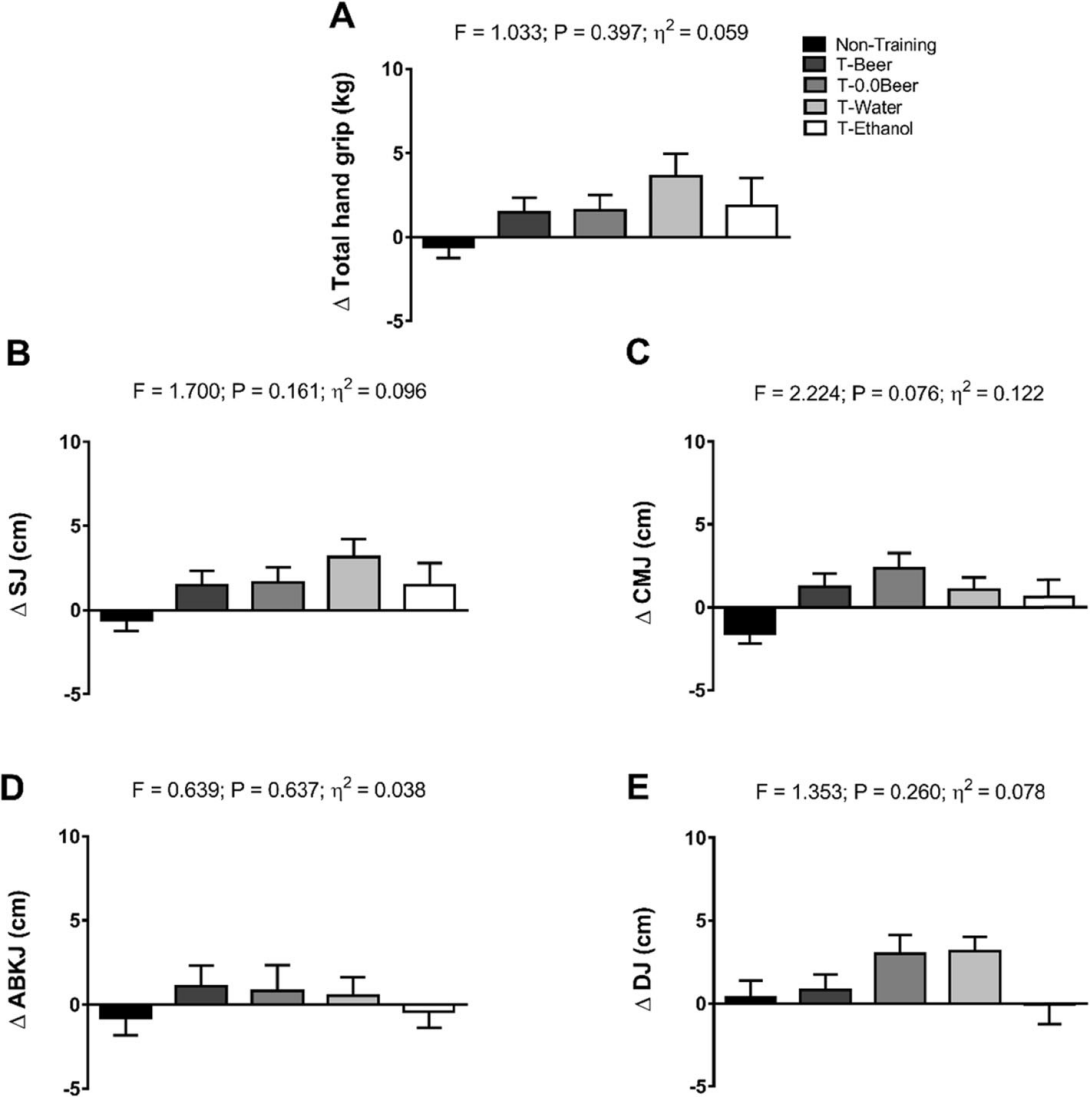
Changes in total hand grip (**a**), squat jump (**b**), counter-movement jump (**c**), Abalakov jump (**d**), and drop jump (**e**), after the intervention study between the five groups. Data are shown as means ± standard error of the mean. Abbreviations: ɳ2, partial eta squared; SJ, squat jump; CMJ, counter-movement jump; ABKJ, Abalakov jump; DJ, drop jump; T-Beer, the group that performed HIIT and consumed alcohol beer; T-0.0Beer, the group that performed HIIT and consumed non-alcoholic beer; T-Water, the group that performed HIIT and consumed sparkling water; T-Ethanol, the group that performed HIIT and consumed sparkling water with alcohol added

Figure 5 shows changes in muscular strength and power-related variables after the intervention study among the five groups. All the intervention groups similarly improved total hand grip, SJ, CMJ, ABKJ and DJ performance compared with the Non-Training group, with no statistical differences between them (all *p* > 0.05). The results persisted in all cases when sex and age were included as a covariate (see Additional file 3).

## Discussion

This study shows that a 10-week structured and highly demanding exercise intervention improves cardiorespiratory fitness and hand grip strength in healthy adults. This was not influenced by the concurrent daily intake of beer or ethanol in moderate amounts. No significant improvements were found in muscular power variables. Therefore, moderate and daily beer consumption accompanying meals, like in the present study, appears not to be an issue of concern affecting physical fitness parameters such as cardiorespiratory fitness and muscular strength after a HIIT program in healthy young adults.

Numerous studies have reported a robust evidence that HIIT induces improvements in cardiorespiratory fitness [5–8]. In a recent meta-analysis, Weston et al. [7] concluded that a low-volume HIIT produces moderate improvements in the VO_2_max. of active non-athletic and sedentary subjects. These findings concur with those obtained by Milanović et al. [8], who reported in their systematic review and meta-analysis that HIIT elicits improvements in the VO_2_max. of healthy, young to middle-aged adults, showing greater gains following HIIT than with continuous endurance training. In line with this, we showed that the T-0.0Beer and the T-Water groups increased their VO_2_max. in absolute and relative terms after a 10-week HIIT intervention program. Thus, our results are of great value for public health due to the strong and positive association between better VO_2_max. and reduced morbidity and mortality risk [1, 3], which improves health, well-being and exercise performance [15–18].

Although most research has focused on the relationship between cardiorespiratory fitness and health outcomes, some studies have recently concentrated on muscular fitness. Recent researches have shown that high levels of muscular fitness are associated with a decreased cardiovascular risk and it has also been favorably correlated with improved bone health, enhanced self-esteem, and decreased adiposity [3]. A recent study found that a 12-week HIIT intervention, using the body weight as load, improved muscle strength in young women [54]. These results concur with those obtained by Amaro-Gahete et al., who found that a 12-week body-weight HIIT intervention improved extension and flexion peak torque and hand grip strength in middle-aged adults [55]. The present findings suggest that a 10-week HIIT intervention improves muscular strength (~ 5%, in the T-0.0Beer and the T-Water groups), measured by the hand grip test. On the other hand, Weston et al. [7] suggested that a low-volume HIIT has an unclear effect on muscular power that could be, at most, either a moderately beneficial or a mildly harmful effect. Our results showed no statistically significant increases from 5 to 12% in the three jump types of the Bosco test, showing lighter improvements in ABKJ (1.8% for the T-0.0Beer group, and 5.6% for the T-Water group) after the 10-week of HIIT intervention.

The majority of literature has been unable to establish a significant cause-effect relationship of alcohol with aerobic or anaerobic performance. While some studies have not found significant consequences of alcohol for submaximal endurance performance [56, 57], other research have suggested that alcohol is detrimental to endurance performance [19, 58]. In line with this, our results showed that a daily but moderate consumption of beer, or the equivalent ethanol amount, did not have deleterious effects on cardiorespiratory fitness-related variables, since the T-Beer and the T-Ethanol groups showed similar improvements in VO_2_max. in absolute (17% for the T-Beer and 17.7% for the T-Ethanol) and in relative terms (16.3 and 16.8%, respectively) in relation to the free-alcohol intervention groups. Actually, it is under debate whether the possible beneficial effects of fermented beverages depend on the alcoholic or non-alcoholic components. A narrative review concluded that the protective effects resulting from the moderate consumption of wine or beer are due to both their alcohol and polyphenol components [59]. Some researchers have investigated the impact of acute alcohol consumption on skeletal muscle recovery and its effects on protein synthesis [28]. Poulsen et al. [60] suggested that muscular strength was not affected by moderate alcohol intoxication in healthy subjects. Our results agree with these findings since no detrimental effects were found in the T-Beer and T-Ethanol groups; contrarily to expected, these alcohol-consuming groups showed increases of ~ 3.5 kg in hand grip strength, but without statistically significant differences. On the other hand, it has been reported that large amounts of alcohol consumed immediately after prolonged exercise were associated with impairments of carbohydrates and lipid metabolism, which suggests an indirect deleterious effect of alcohol on muscle glycogen synthesis [22]. However, some authors found that low doses of alcohol ingested after strenuous damaging exercise had no impact on the exercise-induced muscle-damage losses in muscular performance [61], also that alcohol could have little or no effect on the resynthesis of muscle glycogen after exercise in male athletes [28]. Further, our previous results showed that a daily consumption of moderate amounts of beer or alcohol did not impair the gain of lean mass after a 10-week HIIT intervention [62]. Interestingly, the T-Beer, the T-0.0Beer and the T-Water women groups showed improvements with no statistical significance in SJ, Abalakov, and DJ, while the T-Ethanol group showed a decrease in all of them, although with no statistical significance. Similar impairments were found in CMJ for the T-Ethanol men group. Contrary to our results, Levitt et al. [63] found that consuming alcohol after strenuous-eccentric resistance exercise did not affect performance recovery for young recreationally resistance-trained women. Further, these authors [31] found that consuming a moderately high dose of alcohol following strenuous resistance exercise did not affect recovery of vertical jump height in resistance-trained men. In line with our findings, Barnes et al. [30] found that consuming a moderately high dose of alcohol (1 g ethanol kg^− 1^ body mass) after eccentric exercise affected negatively the strength recovery in healthy men. The results of the present study are inconclusive since no significant improvements in muscle power have been found in the other intervention groups. Further studies are therefore required to examine the effectiveness of HIIT self-load program on muscular power and the possible concomitant effect of alcohol intake. In addition, future studies are needed to clarify if alcohol consumption affects differently women and men.

Although our study has demonstrated that a 10-week HIIT intervention can elicit improvements in muscular strength as well as cardiorespiratory fitness and that these improvements are not mitigated by the intake of beer or ethanol, it presents some limitations. Firstly, the sample size was relatively small to study the influence of different alcohol beverages in moderate amounts during an exercise-training intervention on physical-fitness variables. Considering that we compared a total of four different types of beverages ingested, future studies may replicate this study in larger clinical trials. Secondly, the participants included in our study are young, moderately active, healthy adults. Further research is required to clarify the effects on other populations such as older adults, sedentary, overweight or sport/athlete people; also, future studies are needed to clarify the effects in different atmospherics’ conditions (i.e., lower or higher temperature). Thirdly, although physical activity levels were registered before and after the intervention program, they were subjectively assessed / self-reported. Finally, participants were not purely randomized, basically due to ethical considerations, and, given the organoleptic characteristics of the different types of beverages, a real double-blind design, placebo controlled for alcohol, was not possible.

## Conclusions

This study shows that a 10-week structured and highly demanding exercise intervention improves cardiorespiratory fitness and hand grip strength in healthy adults. This was not influenced by the concurrent intake of beer in moderate amounts. Although we did not find deleterious effects of post-exercise alcohol consumption neither on cardiorespiratory fitness nor on muscular strength, the use of alcohol after strenuous exercise should be managed carefully. Therefore, more information is required if recommendations on appropriate alcohol use during the post-event period are to be made.

## Supplementary information

**Additional File 1: **Changes in maximum oxygen uptake (VO2max.) in absolute (A for men and B for women) and relative terms (C for men and D for women), maximal heart rate (E for men and F for women), and total test duration (G for men and H for women), after the intervention study between the five groups. Significant differences between groups applying an analysis of covariance adjusting for baseline values with post hoc Bonferroni-corrected t-test are indicated as: * *p* < 0.05 ** *p* < 0.01. Data are shown as means ± standard error of the mean. Abbreviations: ɳ2, partial eta squared; T-Beer, the group that performed HIIT and consumed alcohol beer; T-0.0Beer, the group that performed HIIT and consumed non-alcoholic beer; T-Water, the group that performed HIIT and consumed sparkling water; T-Ethanol, the group that performed HIIT and consumed sparkling water with alcohol added.

**Additional File 2:** Changes in total hand grip (A for men and B for women), squat jump (C for men and D for women), counter-movement jump (E for men and F for women), Abalakov jump (G for men and H for women), and drop jump (I for men and J for women), after the intervention study between the five groups. Data are shown as means ± standard error of the mean. Abbreviations: ɳ2, partial eta squared; SJ, squat jump; CMJ, counter-movement jump; ABKJ, Abalakov jump; DJ, drop jump; T-Beer, the group that performed HIIT and consumed alcohol beer; T-0.0Beer, the group that performed HIIT and consumed non-alcoholic beer; T-Water, the group that performed HIIT and consumed sparkling water; T-Ethanol, the group that performed HIIT and consumed sparkling water with alcohol added.

**Additional file 3:** Changes in physical fitness outcomes adjusted by baseline values (Model 1) adjusted by baseline values and sex (Model 2), by baseline values and age (Model 3).

## Data Availability

All data generated or analyzed during this study are included in this published article [and its supplementary information files].
