# Supplementary figures and images for: Influence of daily beer or ethanol consumption on physical fitness in response to a high-intensity interval training program. The BEER-HIIT study
Source: J Int Soc Sports Nutr. 2020 May 27;17:29. doi: 10.1186/s12970-020-00356-7 (PMC7254771; doi:10.1186/s12970-020-00356-7)

**
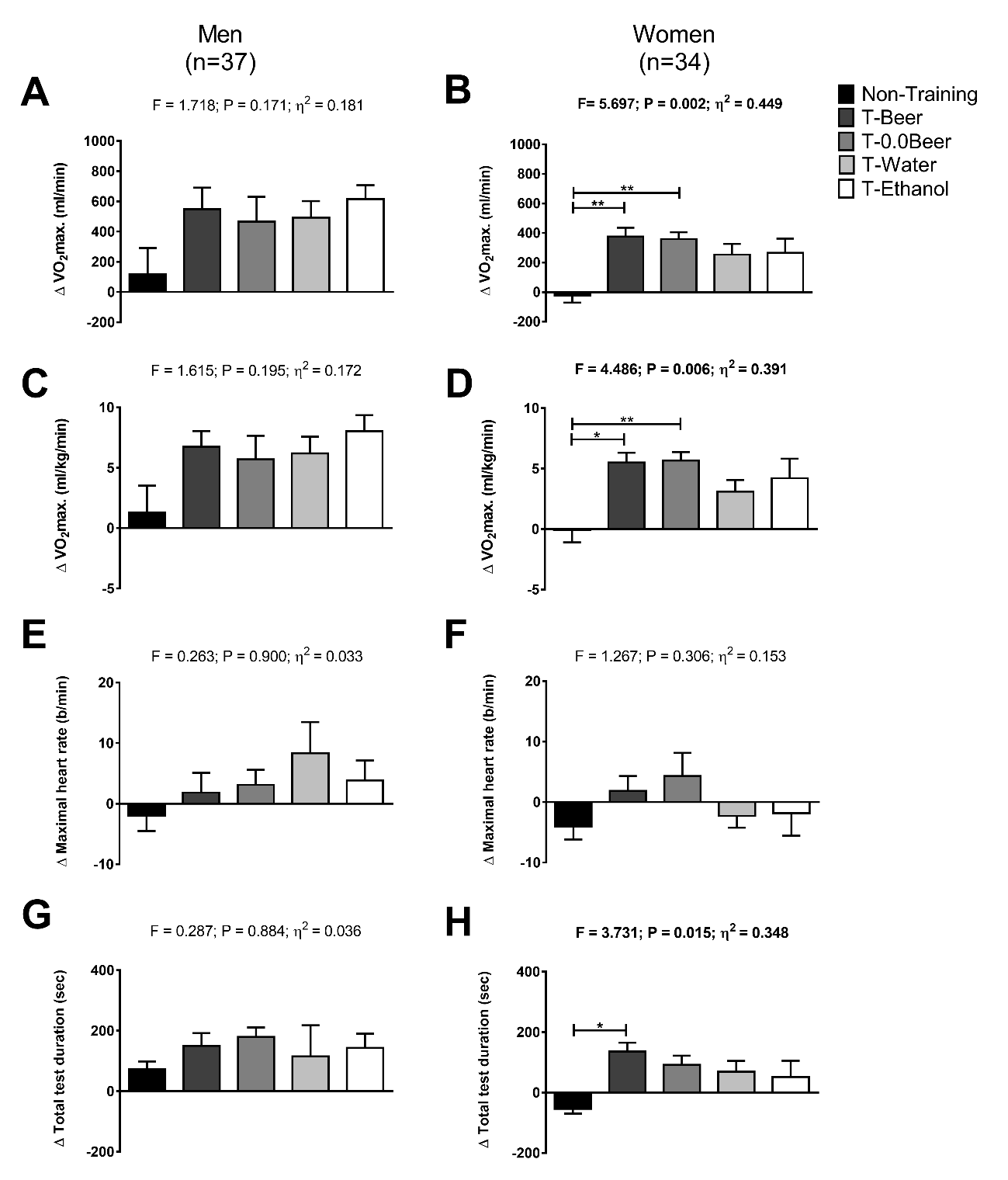
**

**Additional file 1**

Supplement: Supplementary file 1 — Additional File 1: Changes in maximum oxygen uptake (VO2max.) in absolute (A for men and B for women) and relative terms (C for men and D for women), maximal heart rate (E for men and F for women), and total test duration (G for men and H for women), after the intervention study between the five groups. Significant differences between groups applying an analysis of covariance adjusting for baseline values with post hoc Bonferroni-corrected t-test are indicated as: * p < 0.05 ** p < 0.01. Data are shown as means ± standard error of the mean. Abbreviations: ɳ2, partial eta squared; T-Beer, the group that performed HIIT and consumed alcohol beer; T-0.0Beer, the group that performed HIIT and consumed non-alcoholic beer; T-Water, the group that performed HIIT and consumed sparkling water; T-Ethanol, the group that performed HIIT and consumed sparkling water with alcohol added. [file 12970_2020_356_MOESM1_ESM.docx]

**
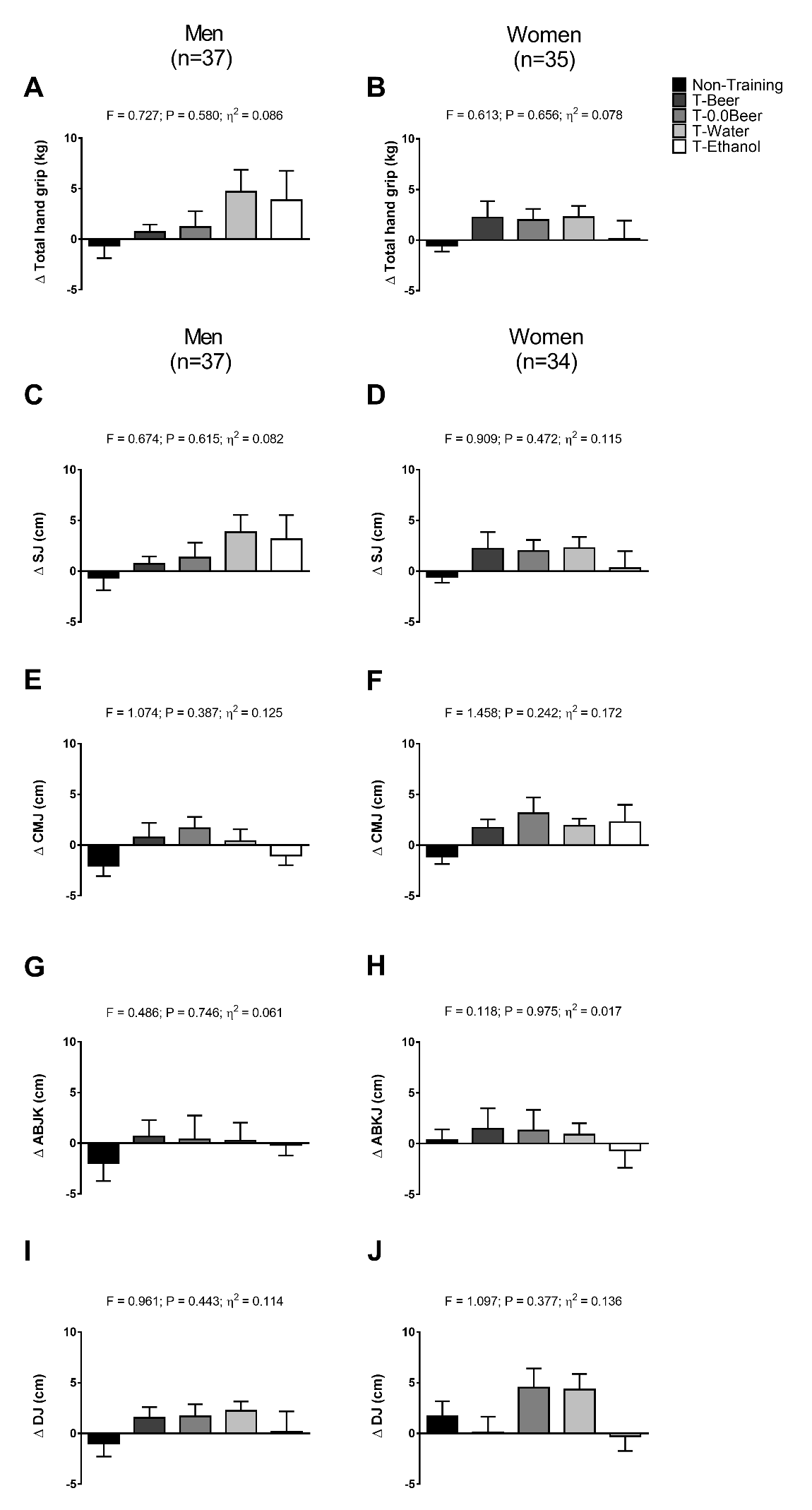
**

**Additional file 2**

Supplement: Supplementary file 2 — Additional File 2: Changes in total hand grip (A for men and B for women), squat jump (C for men and D for women), counter-movement jump (E for men and F for women), Abalakov jump (G for men and H for women), and drop jump (I for men and J for women), after the intervention study between the five groups. Data are shown as means ± standard error of the mean. Abbreviations: ɳ2, partial eta squared; SJ, squat jump; CMJ, counter-movement jump; ABKJ, Abalakov jump; DJ, drop jump; T-Beer, the group that performed HIIT and consumed alcohol beer; T-0.0Beer, the group that performed HIIT and consumed non-alcoholic beer; T-Water, the group that performed HIIT and consumed sparkling water; T-Ethanol, the group that performed HIIT and consumed sparkling water with alcohol added. [file 12970_2020_356_MOESM2_ESM.docx]
