# Supplementary material for: Influence of daily beer or ethanol consumption on physical fitness in response to a high-intensity interval training program. The BEER-HIIT study
Source: J Int Soc Sports Nutr. 2020 May 27;17:29. doi: 10.1186/s12970-020-00356-7 (PMC7254771; doi:10.1186/s12970-020-00356-7)
Supplement: Supplementary file 3 — Additional file 3: Changes in physical fitness outcomes adjusted by baseline values (Model 1) adjusted by baseline values and sex (Model 2), by baseline values and age (Model 3). [file 12970_2020_356_MOESM3_ESM.docx]

**Additional file 3**. Changes in physical fitness outcomes adjusted by baseline values (Model 1) adjusted by baseline values and sex (Model 2), by baseline values and age (Model 3).

|  | | Analysis of covariance P value | | | | |
| --- | --- | --- | --- | --- | --- | --- |
|  | | | F | P | η^2^ | |
| VO_2_max. (ml/min) | Model 1 | | **4.262** | **0.004** | | **0.208** |
|  | Model 2 | | **4.671** | **0.002** | | **0.226** |
|  | Model 3 | | **5.391** | **0.001** | | **0.252** |
| VO_2_max. (ml/kg/min) | Model 1 | | **4.418** | **0.003** | | **0.214** |
|  | Model 2 | | **4.381** | **0.003** | | **0.215** |
|  | Model 3 | | **5.511** | **0.001** | | **0.256** |
| Maximal heart rate (b/min) | Model 1 | | 0.856 | 0.495 | | 0.050 |
|  | Model 2 | | 0.853 | 0.497 | | 0.051 |
|  | Model 3 | | 1.090 | 0.369 | | 0.064 |
| Total test duration (sec) | Model 1 | | 1.760 | 0.148 | | 0.098 |
|  | Model 2 | | 1.803 | 0.139 | | 0.101 |
|  | Model 3 | | 1.800 | 0.140 | | 0.101 |
| Total hand grip (kg) | Model 1 | | 1.033 | 0.397 | | 0.059 |
|  | Model 2 | | 1.176 | 0.330 | | 0.067 |
|  | Model 3 | | 0.913 | 0.463 | | 0.053 |
| Squat jump (cm) | Model 1 | | 1.700 | 0.161 | | 0.096 |
|  | Model 2 | | 1.540 | 0.201 | | 0.089 |
|  | Model 3 | | 1.582 | 0.190 | | 0.091 |
| Counter-movement jump (cm) | Model 1 | | 2.224 | 0.076 | | 0.122 |
|  | Model 2 | | 2.257 | 0.073 | | 0.125 |
|  | Model 3 | | **4.793** | **0.002** | | **0.223** |

| Additional file 1 continued | | | | |
| --- | --- | --- | --- | --- |
| Abalakov jump (cm) | Model 1 | 0.639 | 0.637 | 0.038 |
|  | Model 2 | 0.541 | 0.706 | 0.033 |
|  | Model 3 | 0.726 | 0.577 | 0.044 |
| Drop jump (cm) | Model 1 | 1.353 | 0.260 | 0.078 |
|  | Model 2 | 1.161 | 0.336 | 0.069 |
|  | Model 3 | 1.338 | 0.266 | 0.078 |
